# The effectiveness of current informative material in improving awareness and opinion of undergraduate students towards organ donation: a comparative, randomized survey study

**DOI:** 10.6061/clinics/2019/e743

**Published:** 2019-04-16

**Authors:** Eduardo Riccetto, Ilka Santana de Fátima Ferreira Boin

**Affiliations:** I Faculdade de Ciencias Medicas, Universidade Estadual de Campinas, Campinas, SP, BR; II Divisao de Doencas do Figado, Trato Biliar e Transplante Hepatico, Faculdade de Ciencias Medicas, Universidade Estadual de Campinas, Campinas, SP, BR; III Servico de Transplante de Figado, Hospital das Clinicas, Campinas, SP, BR

**Keywords:** Transplantation, Education, Tissue and Organ Procurement

## Abstract

**OBJECTIVES:**

Despite the contribution of awareness campaigns to the rise of organ donation rates in Brazil, younger folks are subject to few awareness actions. Records on the effect of informative campaigns at improving opinion and knowledge of undergraduates about organ donation are scarce. This study aimed to assess the effect of informative material about organ donation on changes in the trend of answers to a questionnaire compared to the answers of a control group.

**METHODS:**

Two randomized groups were compared, receiving the same standardized questionnaire. One group was supplied informative material on the subject, while the other was not. The questionnaire was sent to undergraduate students from two Brazilian universities. Statistical analysis was performed using Student's t-test, Chi-square test and multinomial regression tests. Adopted significance was 5%.

**RESULTS:**

There were 739 responses to the questionnaire. Mean age was 22 years, with a majority of women. Six of 14 questions displayed a change in the answer pattern of the experimental group compared to controls (*p*<0.05). Opinion on organ donation had changes in 2 of 7 analyzed questions (*p*<0.05). Knowledge on the subject had a shift in answer patterns in 4 of 7 questions. Regression demonstrated 3 items that were not influenced by respondents' age.

**CONCLUSION:**

There is controversy regarding the benefit of exposure to informative material. Negative changes were noted in the trust in transplantation as a safe treatment. Positive results regarding technical knowledge were obtained. Better results may be obtained by designing informative material tailored towards the student's specific concerns.

## INTRODUCTION

Individuals from 18 to 35 years of age make up approximately a fifth of all registered post mortem donors in Brazil 1. They also comprise the age group that is most willing to donate organs after death 1. Yet, awareness campaigns about organ donation are rarely aimed at the younger public.

Despite displaying a large willingness to donate in this age group – up to 89% of those inquired stated that they intended to donate their organs 2,3 – young adults most of the time fail to become post-mortem donors upon their deaths 4, possibly due to miscommunication with family members, divergence of opinion on the matter or lack of knowledge on how to properly state the will to become and organ donor 5. Literature evidence on the knowledge of undergraduates about organ donation shows that educational systems fail to provide information to students on this matter 6,7.

The failure to convert willingness into donation is evidenced by the number of effective donors nationwide in 2017 4. The value is much lower than the intent to donate, mostly due to family members denying extraction of the deceased's organs. In 2017, the rate of denial to donate reached 42% of all family interviews of potential donors. Regionally, the number drops slightly but is still high at 37% of denial to donate 4.

Upon such impacts caused by misinformation in potential donation rates – namely along younger demographic groups – it is crucial to try to establish the effectiveness of current informative materials on organ donation.

Such act is essential not only to assess its adequateness to mitigate current doubts about the matter displayed by the younger public but also to gauge its capacity to inform one about the technical aspects of transplantation displaying a favorable opinion about the process.

This study aimed to assess the effect of informative material about organ donation to shifts in the trend of answers undergraduates to a questionnaire, comparatively to the answers of a control group.

## MATERIALS AND METHODS

The authors e-mailed a 20 multiple-choice question questionnaire to all 17,895 undergraduate students from two Brazilian universities (Sao Leopoldo Mandic Faculty – SLMANDIC - and Campinas State University - UNICAMP). The questionnaire assessed opinions and knowledge on organ donation. A consent form, previously approved by the Ethics Committees of Human Experimentation of the enrolled universities, was also sent to all participants.

Individuals were randomly assigned to either the experimental or control groups by the SurveyMonkey software program (SurveyMonkey, San Mateo, CA, USA). Randomization was performed in a 1:1 manner and included only individuals who accepted the consent form. Non-responders were not randomized, but individuals with incomplete responses to the questionnaire were. Researchers were blinded to the randomization and had no control over the process.

Members of the experimental group were sent informative material about organ donation before starting the survey. The material was collected from non-profit organizations that promote organ donation in Brazil: the National Council of Donor Families 8 and the Brazilian Association for Organ Donation 9. Both materials are of free access and distribution and have been used in campaigns about organ donation in the past.

The referred questionnaire comprised 7 items assessing data on the respondent (gender, age, educational level of closest relatives, religion, field of study, acquaintance with organ or donor recipient relatives and the intention to be and organ donor). Another 7 items assessed the respondent's opinion on organ donation, followed by 6 more items assessed knowledge on the subject – with scenarios raging from which type of deceased individuals qualify to be organ donors, to the correct way to express one's will to become an organ donor upon death. Individual questions are displayed in Table 1.

The distribution of age between study groups was assessed by the t-student test for independent samples. Other demographic data (gender, religion, educational level of closes relative, field of study, willingness to donate and proximity with an organ donor or receptor) were assessed by chi-square tests.

The impact of the informative material on the answer patterns of each study group was established by analyzing each question separately, also by chi-square tests. Questions that presented significance in the chi-squared tests were also submitted to multinomial logistic regressions accounting for age.

Response patterns were also compared according to collected demographic data. In these comparisons, respondents from both groups were analyzed together, being compared only by their demographic profile.

The adopted significance value was 5% for all tests. The Statistical Analysis System for Windows, version 9.4, was employed for analysis (SAS Institute Inc., Cary, NC, USA).

### Ethics

This study was aproved by the Ethics cometee's of both participating universities. All participants were provided with a consent form, which had to be signed in order to participate in the study.

## RESULTS

**Figure 1:**
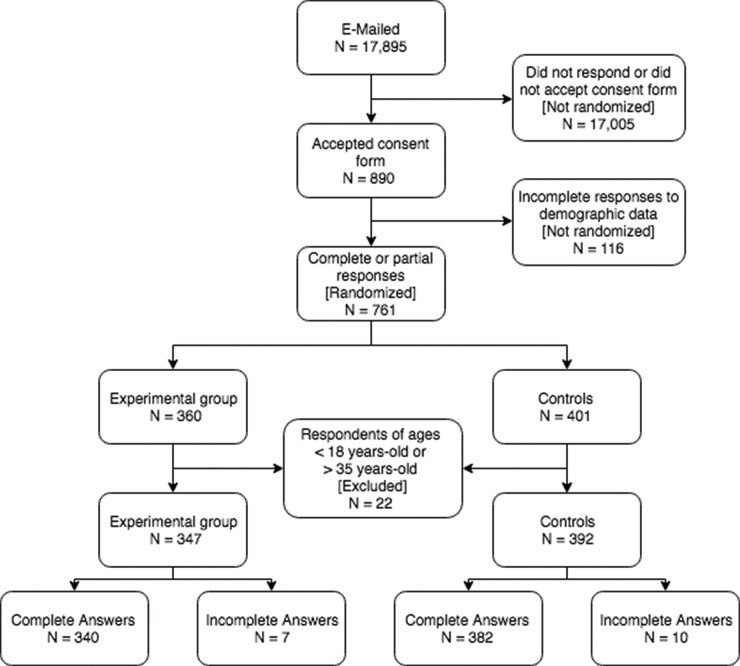
Selection of valid responses and randomization of participants in experimental and control groups.

Seven hundred and twenty-two complete answers to the questionnaire were received, of which 340 were from the experimental group and 382 were from the controls. There were also 17 partial responses, 7 from the experimental group and 10 from controls. Non-responders were not randomized and not included in the analysis because randomization was performed solely upon completing demographic data collection. Individuals younger than 18 or older than 34 years of age (N=24) were also excluded from the analysis because the focus of this study is the younger demographics in the university setting. The way respondents were distributed is shown in Figure 1, and the number of valid answers for each question in both study groups can be seen in Table 1.

The mean age of respondents was 22.15 years old, with a median of 21. Age was distributed normally, as denoted by a standard deviation lower than 4 years and very close values of means and medians. Controls had a lower median age than the experimental group when compared by the student t-test (*p*=0.043), most likely due to the method of randomization being 1:1 and not accounting for compensation for any specific demographic variables in the sample. The authors observed no hazardous matter that could influence such distribution. This age difference was disregarded because it represented only 0.48 years and was not practically distinguishing between the study groups.

The majority of respondents were women (65.1% of total), which is an accurate gender distribution for the academic environment 10. Controls had a higher number of women than the experimental group, as demonstrated by chi-square tests (*p*=0.063) (*p*<0.05).

Field of study, educational level of closest relative, religion and acquaintance with an organ donor or receptor relative were evenly distributed between both groups (*p*>0.05). Overall, respondents were mainly from the formal and applied sciences field (48.5%), had close relatives with a university degree (44%), were Catholics (30%) and had no acquaintance to organ donor or receiver relatives (90%). Detailed demographic data are displayed in Table 1.

Six of the 14 analyzed items in the questionnaire presented changes in the answer patterns of the experimental group when compared to controls. The respective p values for the chi-square tests in each question are displayed in Table 2.

The experimental group demonstrated different answer patterns in regard to opinion and knowledge about organ donation when compared to controls in 3 out of 7 questions on the section that assessed opinion and 3 out of 7 in the section that assessed knowledge.

The opinion on organ donation showed differences in the experimental group's answer pattern compared to controls in items 12 (*p*=0.021) and 13 (*p*<0.001). The knowledge of the process of organ donation showed differences in the experimental group's answer patterns in items 14 (*p*<0.0001), 18 (*p*<0.0001) and 20 (*p*=0.006) when compared to controls.

A multinomial logistic regression for age was performed in the items that showed significance in the chi-square tests, and 3 out of six showed maintained significance and no influence of respondents' age the answer patterns: 12 (*p*=0.021); 16 (*p*=0.016); 20 (*p*=0.006) (Table 2). Items 13, 14 and 18 demonstrated that age influenced answer choices but still maintained statistical significance.

Women tended to express a more favorable opinion about organ donation when compared to men. Additionally, the majority of respondents stated that religious choice did not influence their beliefs on organ donation (85% in item 11 of the questionnaire).

Comparing respondents by age groups, broad fields of study and educational level of relatives showed no shift in answer patterns. A high educational level background, which tended to be one of such important factors for adults 11-14, did not influence the willingness of respondents to donate.

Although age was unequally distributed across study groups, its low absolute mean difference has led the authors to consider all variables equally distributed, as randomization was performed correctly. In doing so, we can attribute the changes of answer patterns between the two groups to the presence of absence of the offered informative materials – which is a strong indication of their effectiveness.

## DISCUSSION

Data gathered from this survey study demonstrated important features of the use of the current informative materials on the field of organ donation, namely, its particular strengths and weaknesses. The large number of participants from varying academic backgrounds, religions, and socioeconomic conditions contributed to the quality of the gathered data.

The changes observed between the two study groups in regards to opinion related to organ donation show a possibly grave flaw of the provided informative material: answer patterns in the experimental group were less trusting in the clinical value of transplantation compared to controls.

Additionally, fewer respondents in the experimental group declared that transplantation was a safe and effective treatment method (item 12). The authors attribute such decline in opinion to both a sense of cautiousness associated with the expose to information about organ donation and a possible flaw in the provided informative material in emotionally appealing towards the reader. This behavior was demonstrated to be unrelated to respondents' ages upon multinomial regression.

Differences in the preferred method to reach reliable information about organ donation between study groups were denoted in item 13: members of the experimental group more often chose ‘Internet' as the main instrument of information about organ donation, against other means of communication. Upon multinomial regression, age was found to influence response patterns while maintaining statistical significance: older individuals tended to prefer academic books and television rather than the Internet to inform themselves about organ donation.

In regards to the changes in answer pattern observed in the section related to technical knowledge about organ donation, the exposure was truly beneficial to the experimental group. Respondents from the experimental group stated that they were well informed about the matter of organ donation much more than respondents from the control group (question 14). Additionally, respondents proved to be qualified to know how to properly state the will to be an organ donor in the future and to educate others to do so properly (question 18).

Multinomial regression of items 14 and 18 demonstrated that age did have an influence on the answer patterns: in item 14, older respondents tended to more often report being sufficiently informed about organ donation, while in item 18, younger respondents tended to more often answer correctly to the question and proving to be capable of orienting someone on how to declare their will to donate organs.

The average age of the sample was quite low – only 23 years old. This finding is comparable to studies in other university settings nationally and worldwide 10,15,16. The low mean age of respondents could be influential in the shaping of response patterns, as this age group presents with different values regarding organ donation compared to older populations. Its impact is better assessed in the limitations of this study.

Women made up 65% of the total number of participants in our study, which is comparable with gender distributions in other works in university settings 10,15-17. Other studies that addressed the general population presented a more homogeneous distribution between genders 1,2,5,11-13,18,19.

Such demographic disposition may be of special interest due to literature evidence suggesting that women are usually more willing to authorize organ donations of deceased relatives or become organ donors themselves 12,18. This evidence is in par with the findings in our study, in which women tended to have a more positive response on the will to become an organ donor. Vijayalakshmi et al. 13, however, displayed different results in their studies conducted in India – women were more reluctant than men to sign a donor's card, which may be explained by local religious and cultural practices.

We evidenced a predominance of respondents with close relatives with university degrees. Many studies in the literature had samples with individuals with university degrees or undergraduate university courses 2,3,6,12,15-17. Chung et al. 19 addressed individuals in primary and middle school, and others have focused on individuals outside an academic setting 1,5,11,13,18,20,21.

Although having a majority of respondents from a relatively high socioeconomic and educational standpoint, such status did not influence the respondent's willingness to donate in our study, which might be due to the importance of one being in a university background having greater influence than the educational background of parents or relatives.

Most studies, from both Europe and Asia, had samples with a majority of religious individuals 1,13-15,22. Terbonssen et al. 17 showed that among German medical students, a higher willingness to donate was associated, in many aspects, with a lack of religion, especially towards the belief in afterlife. Vijayalakshmi et al. 13 displayed results in which half of the analyzed sample thought most religions opposed organ donation.

Conversely, in our study, most participants displayed some religious belief, mostly Christian Catholicism. However, when asked about the influence of religious beliefs on the decision to be an organ donor, 85% of participants (regardless of being on the experimental or control group) stated that such decisions were not influenced by religious beliefs.

Studies assessing a sample's general knowledge about organ donation 1,2,11,13,14,16,17 have demonstrated misinformation on the same subjects as the control group in our study. Terbossen et al. 17 showed that German medical students who did not carry a donor's card feared feeling pain during organ extraction procedures.

Such misconceptions were also found in our study (item 16), albeit having a much lower prevalence in the group exposed to informative material (*p*<0.001). The fear of not being dead upon organ extraction showed a significant decrease in the previously informed participants compared to controls. Such behavior was maintained even upon multinomial regression accounting for age.

Despite the quality of the gathered data and its comparability to other works, this study had relevant limitations. One of the most prominent of them is the use of a non-validated questionnaire to assess the impact of informative material. This was due to the lack of a standardized questionnaire for such purposes in the current literature. The fact that the material is free for access over the Internet is also a limitation to the study.

Other limitations of the study are the inheritant traits of online, impersonal questionnaires. The rate of response of this method of distribution tends to be low, reaching approximately 5 to 6% response rates 23. Be that as it may, the demographic profile of respondents was comparable to the usual profile seen in university environments 10.

Respondent gender might have influenced the outcomes in our study, as women are more favorable to organ donation, as previously shown. However, the majority of women in our sample reflect the gender distribution in the university environment as whole, where women comprise the majority of undergraduates 10,17.

Based on our findings and their comparison to the existing works in the literature, we can conclude that the informative material that participants were offered was dichotomous in its effect – providing excellent technical knowledge about the donation process but causing harmful impacts in the opinion towards organ donation.

Results displayed the important untapped potential in establishing better awareness of younger folks towards the matter of organ donation, as they may forward such knowledge as future trendsetters, or even use the acquired information when faced with personal situations that involve organ donation.

Upon such evidence and untapped potential, it is clear that better and more comprehensive informative campaigns have to be developed and carried out, with regards to the specific concerns and doubts of individuals from each age group and its demographic characteristics, especially towards improving opinion and emotionally conveying a positive message about organ donation.

## AUTHOR CONTRIBUTIONS

Riccetto E was responsible for the study design, data collection, analysis/interpretation, statistics, manuscript drafting. Boin IS was responsible for the study design, data analysis/interpretation, manuscript drafting and critical revision.

## Figures and Tables

**Table 1:** Comparative analysis performed on the answers of the experimental and control groups to the standardized questionnaire. Chosen statistical test for each question is displayed after the *p* value in each row: t-stud=t-Student; C=Chi squared.

Question	N	Answers from control (C) and experimental (E) groups			*p*
		Answer	C	E	
1. Birth date	739	-	Mean age: 21.9 years old	Mean age: 22.4 years old	**0.044 (t-Stud)**
2. Gender	739	Male	124 (31.6)	127(36.6)	0.116 (C)
		Female	267 (68.1)	214 (61.7)	
3. Field of study	739	Humanities and social sciences	108 (27.6)	100 (28.8)	0.846 (C)
		Formal and applied sciences	197 (50.3)	167 (48.1)	
		Biological and health sciences	87 (22.2)	80 (23.1)	
4. Educational level of closest relative	739	Incomplete middle school	21(5.47)	21 (6.1)	0.616 (C)
		Complete middle school	29 (7.3)	18 (5.2)	
		Complete high school	108 (27.6)	107 (30.8)	
		Complete undergraduate	180 (45.9)	150 (43.2)	
		Complete postgraduate	54 (13.8)	51 (14.7)	
5. Religion	739	Catholicism	119 (30.4)	104 (30.0)	0.245 (C)
		Protestant Christianity	43 (11.0)	32 (9.2)	
		Spiritism	42 (10.7)	29 (8.4)	
		Umbanda / Candomblé	7 (1.8)	11 (3.2)	
		Atheism	102 (26.0)	80 (23.1)	
		Others	79 (20.2)	91 (26.2)	
6. Were you or are you acquainted with any relative who received or donated organs?	739	Yes	41 (10.5)	32 (9.2)	0.574 (C)
		No	351 (89.5)	315 (90.8)	
7. Do you intend to donate your organs after death?	739	Yes	353 (90.1)	297 (85.6)	0.119 (C)
		No	5 (1.3)	10 (2.9)	
		Not sure	34 (8.7)	40 (11.5)	
8. Have you ever expressed or intend to express your wish to be an organ donator to a family member?	739	Yes, I have expressed or plan to express my wish	315 (80.4)	281 (81.0)	0.831 (C)
		No, I have not expressed my wish	77 (19.6)	66 (19.0)	
9. You are told that a relative of yours died at the hospital. He or she did not clarify the wish to be an organ donor. Had you been appointed as the one responsible for the decision of whether to donate the organs or not, would you authorize it?	739	Yes	312 (79.6)	253 (72.9)	0.059 (C)
		No	19 (4.8)	29 (8.3)	
		Not sure	61 (15.6)	65 (18.7)	
10. You are told that a relative of yours has been diagnosed with brain death after an accident. He or she expressed the wish to be an organ donor after death. Had you been appointed as the responsible for the decision or whether or not to donate the organs, would you authorize it?	739	Yes	369 (94.1)	319 (91.9)	0.066 (C)
		No	1 (0.3)	7 (2.0)	
		Not sure	21 (5.6)	22 (6.1)	
11. Do your religious principles have any influence over your decision to be or not to be an organ donor?	739	Yes	41 (10.5)	31 (8.9)	0.313 (C)
		No	15 (3.8)	21 (6.1)	
		Not sure	336 (85.7)	295 (85.0)	
12. Do you believe that organ transplantation procedures are an effective and reliable treatment option?	739	Yes	351 (89.5)	300 (86.5)	**0.021 (C)**
		No	1 (0.3)	9 (2.6)	
		Not sure	40 (10.2)	38 (11)	
13. Which of the following media informed you the most about organ donation?	739	Television	102 (26.7)	47 (13.8)	**<0.001 (C)**
		Internet	138 (36.1)	159 (46.8)	
		Radio	0 (0)	0 (0)	
		Booklets from informative campaigns	33 (8.6)	51 (15.0)	
		Books and academic materials	53 (13.9)	36 (10.6)	
		Others	56 (14.7)	47 (13.8)	
14. Would you say that you are sufficiently informed about organ donation?	739	Yes	138 (35.2)	202 (58.2)	**<0.001 (C)**
		No	187 (47.7)	108 (31.1)	
		Not sure	67 (17.1)	37 (10.7)	
15. Would you fear that a relative diagnosed with brain death was not dead?	722	Yes	124 (32.5)	99 (29.1)	0.125 (C)
		No	176 (46.1)	185 (54.4)	
		Not sure	82 (21.5)	56 (16.5)	
16. Would you fear that an organ-donor relative diagnosed with brain death could suffer or feel pain during the procedures or organ extraction?	722	Yes	49 (12.8)	36 (10.6)	**<0.001 (C)**
		No	269 (70.4)	282 (82.2)	
		Not sure	64 (16.8)	22 (6.5)	
17. Would you fear that an organ-donor relative could have his or her body disfigured by the organ extraction procedures?	722	Yes	34 (8.9)	31 (9.1)	0.143 (C)
		No	312 (81.7)	292 (85.9)	
		Not sure	36 (9.4)	17 (5.0)	
18. A relative tells you of his wish to be an organ donor but does not know how to properly record his decision. How would you instruct him?	722	Record in a written and signed document	158 (41.4)	110 (32.4)	**<0.0001 (C)**
		Record in driver’s license or ID card	89 (23.3)	54 (15.9)	
		Record by verbal expression to a relative	118 (30.9)	171 (50.3)	
		Other	17 (4.5)	5 (1.5)	
19. Would you fear that the organs extracted from donors could be sold or used for illegal purposes?	722	Yes	127 (33.2)	135 (39.7)	0.322 (C)
		No	218 (57.1)	176 (51.8)	
		Not sure	37 (9.7)	29 (8.5)	
20. A relative of yours is brought to the hospital in cardiac arrest and is declared dead. He or she expressed a will to be an organ donor upon death. Does this relative qualify to be an organ donor?	722	Yes, for all organs and tissues	27 (7.50)	34 (10.0)	**0.006 (C)**
		Yes, but only for some organs and tissues	201 (52.3)	182 (53.5)	
		No, he / she does not qualify for donation	31 (8.1)	48 (14.1)	
		Not sure	123 (32.2)	458 (22.4)	

**Table 2:** Multinomial logistic regressions accounting for age – Items 13, 14 and 18

Item		*p*	OR	95% CI
Q13^a^				
Booklets from informative campaigns	Intercept	0.004		
	Age	0.158	1.055	0.979-1.137
Books and academic materials	Intercept	0.000		
	Age	0.002	1.114	1.041-1.193
Others	Intercept	0.047		
	Age	0.484	1.026	0.955-1.103
Television	Intercept	0.000		
	Age	0.008	1.084	1.021-1.151
Q14^b^				
Not sure	Intercept	0.373		
	Age	0.718	0.987	0.917-1.062
Yes	Intercept	0.010		
	Age	0.039	1.053	1.003-1.106
Q18^a^				
Record in driver’s license or ID card	Intercept	0.866		
	Age	0.343	1.075	0.926-1.247
Record by a written and signed document	Intercept	0.077		
	Age	0.801	0.981	0.847-1.137
Record by verbally expressing it to a relative	Intercept	0.668		
	Age	0.251	1.089	0.942-1.258

^a^The reference category is Internet.

^b^The reference category is No.
